# Optimization of field setup for organs at risk sparing and beam on time in breast radiotherapy planning for a new volumetric modulated arc therapy based treatment technique

**DOI:** 10.1016/j.phro.2026.101016

**Published:** 2026-06-07

**Authors:** Topi Nykänen, Ville Raatikainen, Aarno Kärnä, Katariina A.H. Näkki, Tuomas Koivumäki

**Affiliations:** a Department of Medical Physics, Hospital Nova of Central Finland, Wellbeing Services County of Central Finland, Hoitajantie 3, 40620, Jyväskylä, Finland; b Department of Physics, University of Jyväskylä (JYU), Survontie 9 C, 40014 Jyväskylä, Finland

**Keywords:** Radiotherapy, Volumetric modulated arc therapy, Breast cancer, Treatment planning

## Abstract

**Background and purpose:**

The limitation of volumetric arc therapy (VMAT) in breast radiotherapy has been the low dose spread, especially to the contralateral side. A new treatment technique which combines VMAT and static angle modulated ports (STAMPs), which mimic intensity modulated radiotherapy (IMRT) fields, could provide a solution for this and lower treatment times.

**Materials and methods:**

The evaluation was done retrospectively on 21 breast cancer patients where two and three arc setups of the new proposed treatment techniques were compared to VMAT plans with four split arcs. The number of STAMPs and the weighting in optimization between the arcs and STAMPs was altered in the plans. This study evaluated the dose-volume histogram parameters and beam on time of this new treatment technique compared to VMAT.

**Results:**

The new technique lowered mean dose of the contralateral breast by 29–62% but increased ipsilateral lung mean doses by 7–16%. Lower number of STAMPs along with lower weighting provided the shortest beam on times. Median beam on times with VMAT were between 131 and 139 s. The fastest setups with the new technique had median times between 110 and 114 s.

**Conclusions:**

Our findings show that the new technique has potential to significantly improve sparing of the contralateral side in breast radiotherapy. In addition, the new technique can lower beam on times compared to VMAT, but this is dependent on the number and weighting of the STAMPs. Lower STAMP weighting was found to be the best option for optimizing treatment time.

## Highlights

• Volumetric modulated arc therapy was combined with static modulated ports.
• The results were compared to volumetric arc therapy in breast cancer radiotherapy.
• Contralateral breast mean dose and beam on times reduced (29–62% and 18%).
• Median beam-on time went from 131 to 139 s to 110–114 s using this new approach.
• Two or three arcs and no more than four static modulated ports are recommended.

## Graphical abstract

**Figure:**
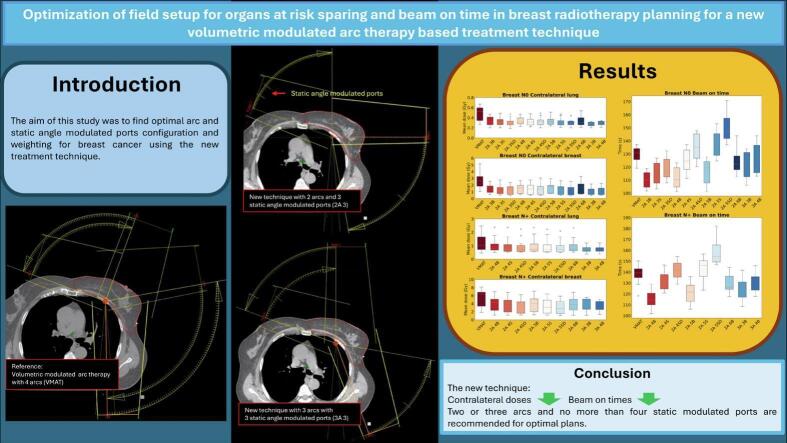

## 1 Introduction

Breast cancer radiotherapy (RT) is commonly delivered using several treatment techniques, including three-dimensional conformal radiotherapy (3D-CRT), field-in-field (FIF), intensity-modulated radiation therapy (IMRT), and volumetric modulated arc therapy (VMAT) [1]. A typical VMAT breast plan utilizes 2 to 5 arcs [1], [2], [3], [4], [5]. Compared to static angle methods, VMAT reduces ipsilateral lung and heart doses as well as treatment times compared to IMRT [5], [6], [7], [8], [9], [10], [11], [12], [13]. A drawback however is that VMAT is associated with increased low-dose exposure to the contralateral lung and breast compared to 3D-CRT or FIF [5], [8], [9].

Previous studies have explored Hybrid-VMAT (HVMAT) approaches combining either 3D-CRT or IMRT with VMAT fields [14], [15], [16], [17], [18], [19], [20], [21], [22]. Compared to VMAT, hybrid techniques have shown reductions in heart, contralateral breast and contralateral lung doses. Hybrid-VMAT was shown to be slower in treatment times than 3D-CRT [17]. Aly et al. [18] reported slightly faster treatment times for HVMAT than VMAT. However, beam on times measured by Lin et al. [22] were considerably longer with VMAT than HVMAT.

A new treatment technique now allows the combining of VMAT arcs with IMRT field mimicking static angle modulated ports (STAMPs), which allow the arc to stop during gantry rotation at the set angles. Initial studies of this approach have shown increases in contralateral sparing and lower beam on time compared to VMAT [23], [24], [25].

The aim of this study was to determine the optimal arc and STAMP configuration for this technique while maintaining planning target volume (PTV) coverage and minimum dose to organs-at-risk (OAR).

## 2 Materials and methods

This study was done retrospectively on a randomly chosen patient cohort of 10 left sided whole breast (N0) and 11 left sided breast patients with nodal involvement (N+). Of the 11 N+ cases, seven were lumpectomies, four had undergone mastectomies and seven patients had the internal mammary chain included in the PTV. The Wellbeing Services County of Central Finland approved the study (updated 23rd April 2025). The patients were imaged with a Somatom Confidence CT (Siemens Healthineers, Germany) in deep inspiration breath-hold (DIBH) with 2 mm slice thickness. The patients were imaged in supine position on an extended wing board with U-grip handle (CQ Medical, USA).

The clinical target volume (CTV) was delineated by a radiation oncologist according to The European Society for Radiotherapy and Oncology (ESTRO) guidelines [26]. A 5 mm uniform PTV margin was added to the CTV. The OARs were automatically contoured in MIM Maestro (MIM software, USA) and verified and corrected by an experienced radiation therapist. The treatment plans were optimized to 40.05 Gy in 15 fractions.

Planning was done in Eclipse (Varian Medical Systems, USA) with the Photon Optimizer algorithm (v18.1). Acuros XB (v18.1) was used for the dose calculation with a 2.5 mm dose matrix. All plans were optimized for a TrueBeam linear accelerator (Varian Medical Systems, USA) equipped with a Millenium 120 multi-leaf collimator. The plans were optimized with 6 MV beam energy. Planning followed the clinical guidelines presented in Supplementary Material A Table S1. A detailed description of the optimization process used for the plans is provided in the Supplementary Material A Planning methodology section.

**Fig. 1:**
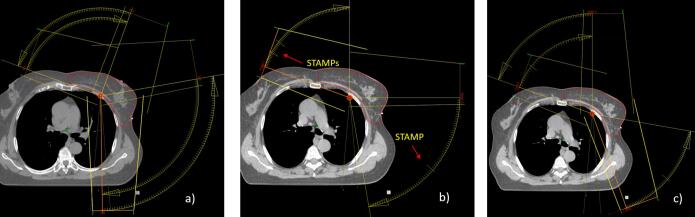
Arc setups for a whole breast case. a) Four split arc VMAT setup. b) Two arc RapidArc Dynamic setup. c) Three arc RapidArc Dynamic setup. RapidArc Dynamic setups have three static angle modulated ports (STAMPs), two in the anterior arcs and one in the posterior arc. In b) and c) the arcs have three larger dashes along the arcs marking the angles of the STAMPs at which the arc rotation stops to allow irradiation from the static angle.

For all patients, a reference VMAT plan was optimized using RapidArc (Varian Medical Systems, USA) with a four split arc configuration (Fig. 1a). The VMAT plans were optimized with a 13 mm skin flash expansion and a 16 mm optimization bolus with a density of −350 HU (Hounsfield Unit). The RapidArc Dynamic (RAD) (Varian Medical Systems, USA) plans were optimized using two or three arcs (Fig. 1b and c). For N0 cases the two arc plans used three to six static angle modulated ports (Table 1). The N+ cases were optimized with four to six tangential STAMPs. The three arc plans were optimized with three and four STAMPs in both cases. For all plans, posterior arcs ranged from approximately 179° [160°-179°] to 80° [60°-90°] and the anterior arcs ranged from approximately 30° [0°-60°] to 290° [290°-295°]. Arc, collimator and STAMP setups are explained in more detail with patient specific arc spans in Supplementary Material Tables S2-S3. The RAD plans were planned using a 13 mm automatic skin flash with an optimization bolus HU of −350. The three, four and five STAMP two arc plans were optimized with the arc/static weighting set to ‘balanced’, ‘static’ and ‘static dominant’ which are options provided by the optimizer. The six STAMP two arc and three arc plans were only optimized with the balanced setting. All RAD plans used the dynamic collimator with the ‘between static angles’ setting, which means that the algorithm optimizes the collimator rotation between user defined STAMPs. All plans were normalized to the mean of the target PTV which had 3 mm of skin removed and was defined as PTV_in_.

**Table 1:** Gantry angles used for the static angle modulated ports (STAMPs) in the different RAD plans.

	Anterior arc	Posterior arc
6 STAMPs	300°, 308°, 316°	123°, 131°, 139°
5 STAMPs	300°, 308°, 316°	123°, 139° or 123°, 131°
4 STAMPs	300°, 308°	123°, 139° or 123°, 131°
3 STAMPs	300°, 308°	131°

Along with PTV_in_ *V*_*95%*_ (%) coverage and *V*_*107%*_ (cm^3^) volumes, various OAR parameters were collected from each plan. The chosen OARs were heart, ipsilateral and contralateral lung, contralateral breast and body. Apart from the body doses, mean dose (Gy) was collected from all OARs, *V*_*16Gy*_ (%) from the heart and ipsilateral lung and *V*_*4Gy*_ (%) from both lungs and contralateral breast. Contralateral breast parameters were unavailable for two N+ patients due to prior bilateral mastectomy. Additionally, body *V*_*20Gy*_ (cm^3^) and *V*_*4Gy*_ (cm^3^) were collected.

The beam on time for all arcs was recorded from the time of pressing the ‘MV Beam on’ button until receiving the signal on the linear accelerator indicating arc completion. In addition to timing, plans were evaluated using electronic portal imaging dosimetry (EPID) followed by gamma analysis. Gamma-analysis was performed on 18 out of 21 VMAT plans and 170 out of 219 RapidArc Dynamic plans. Gamma analysis was performed using absolute normalization, with evaluation criteria 2 mm/3%. Gamma passing rates of ≥95% were considered acceptable.

Wilcoxon signed-rank test was used to evaluate statistical significance (*p* < 0.05) between the VMAT and RAD plans (SPSS v29, IBM, USA). Statistical significance was tested between two arc and three arc plans and between the two three arc plans.

## 3 Results

**Fig. 2:**
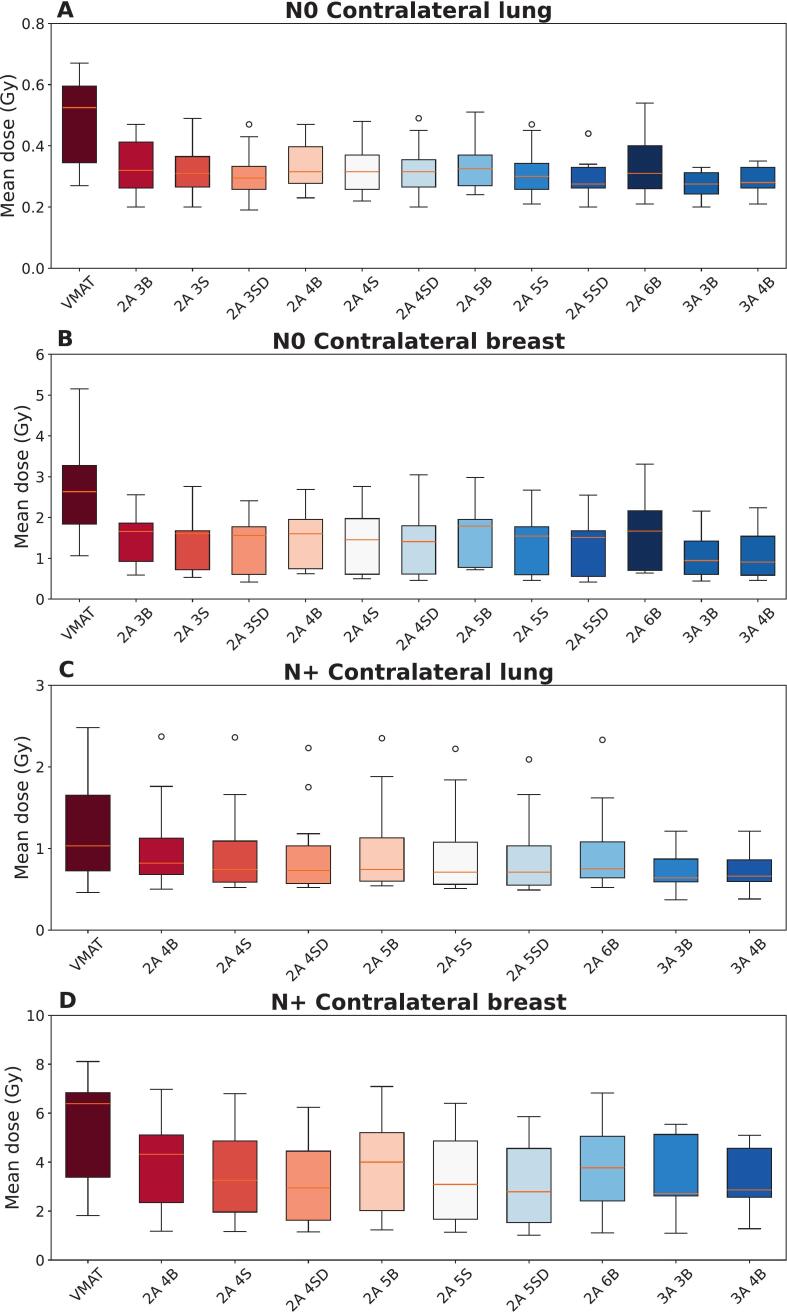
Contralateral lung and contralateral breast mean dose for whole breast (N0) and breast with nodes (N+). Two patients were removed from the N+ patient group for contralateral breast due to bilateral mastectomy (*N* = 9). Here, xA = Arc with x being the number of arcs. B, S and SD refer to the static angle modulated port (STAMP) weighting (B=Balanced, S=Static and SD=Static dominant) with the number in front referring to the number of STAMPs.

**Fig. 3:**
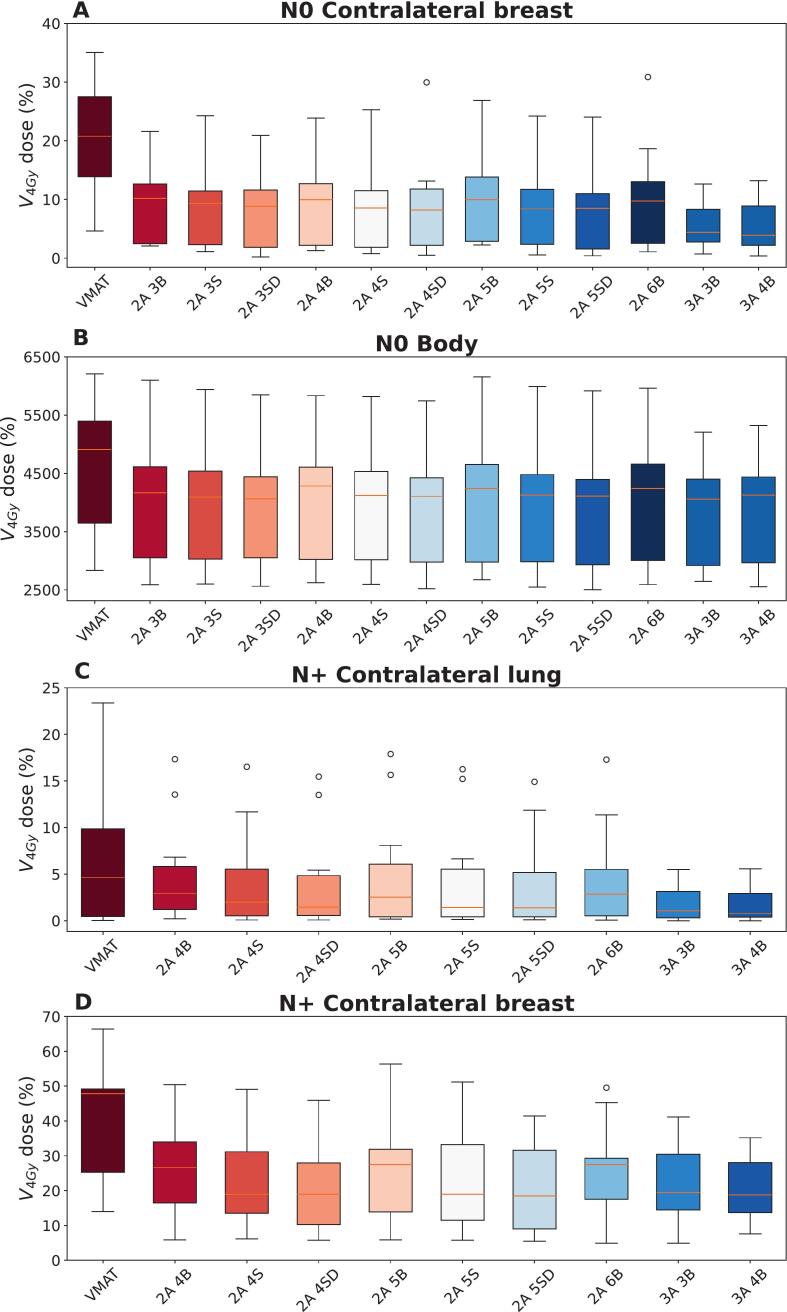
The largest differences seen in low dose *V*_*4Gy*_ between the plans. Contralateral breast and body *V*_*4Gy*_ spread are shown for the whole breast (N0) cases and contralateral lung and breast *V*_*4Gy*_ spread for the breast with nodes (N+) cases. Two patients were removed from the N+ patient group for contralateral breast due to bilateral mastectomy (N = 9). Here, xA = Arc with x being the number of arcs. B, S and SD refer to the static angle modulated port (STAMP) weighting (B=Balanced, S=Static and SD=Static dominant) with the number in front referring to the number of STAMPs.

**Fig. 4:**
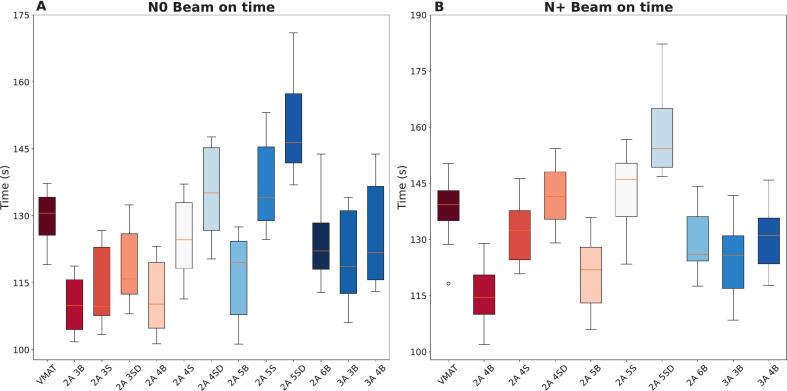
Beam on times for VMAT and the different RAD setups. Here, xA = Arc with x being the number of arcs. B, S and SD refer to the static angle modulated port (STAMP) weighting (B=Balanced, S=Static and SD=Static dominant) with the number in front referring to the number of STAMPs.

The breast N0 patients had a median PTV volume of 1427 cm^3^ [991–2076 cm^3^] and for the breast N+ cases it was 1313 cm^3^ [716–3477 cm^3^]. All plans achieved PTV_in_ *V*_*95%*_ ≥ 95% with no significant differences between VMAT and RAD (Supplementary Material Table S4). Median values and ranges for PTV_in_ V_95%,_ V_107%_ coverage, and OAR parameters are provided in the Supplementary Material Tables S5-S10. All evaluated treatment plans passed the gamma evaluation. Breast N0 VMAT plans had a median of 740 MUs, while breast N+ patients had a median of 781 MUs (Supplementary Material Table S5). Median MUs of RAD ranged from 939 to 1178 and were significantly higher (*p* < 0.05) than in the VMAT plans. The three arc RAD plans had significantly different (*p* < 0.05) MUs compared to the two arc plans in 21 out of 34 setups (Supplementary Material Table S5). Results are presented as boxplots in Fig. 2, Fig. 3, Fig. 4 where the box shows the values from the first quartile to the third quartile and the line in the box shows the median. The whiskers extend from the edge of the box to 1.5 times the inter-quartile range and the circles show the outliers. Example dose volume histograms are presented in Supplementary Material Figs. S11-S12.

In breast N0 plans, median heart *D*_*mean*_ was 1.3 Gy on VMAT and between 1.1 and 1.2 Gy on RAD (Supplementary Material Table S6). Heart mean dose increased in 15 of the 120 N0 RAD plans. Heart *V*_*16Gy*_ remained largely unchanged with largest decreases being 0.4 percentage points (pp).

Ipsilateral lung mean dose increased by 0.2 to 1.0 Gy with RAD compared to VMAT in all but one case (Supplementary Material Table S7). The ipsilateral lung *V*_*16Gy*_ increased by 2.3–2.9 pp. in all RAD plans, while the median increase in lung *V*_*4Gy*_ was between 0.1 and 1.0 pp.

Sparing of the contralateral lung and breast (Fig. 2A & 2B) was more effective with RAD. The median decrease achieved with the different RAD setups in contralateral breast *D*_*mean*_ was between 0.9 and 1.7 Gy compared to VMAT. Contralateral lung *D*_*mean*_ decreased in all RAD cases by 0.1 to 0.4 Gy. Contralateral breast *V*_*4Gy*_ decreased (Fig. 3A) in all but two plans, with the median change being between 8.2 and 15.8 pp. from VMAT to RAD. Contralateral breast mean dose and *V*_*4Gy*_ were lowest with the three arc RAD setup.

Body *V*_*20Gy*_ and *V*_*4Gy*_ (Fig. 3B) decreased with RAD. In all breast N0 RAD plans body *V*_*20Gy*_ was significantly lower (*p* < 0.05) compared to VMAT with the median decrease being between 5.4% and 6.8 %. Median Body *V*_*4Gy*_ decrease was between 12.9% and 17.9%. The largest decreases were in the three arc plans and the two arc plan with five STAMPs with the static dominant setting.

Breast N0 VMAT plans had a median beam on time of 131 s (Fig. 4A & Supplementary Material Table S5). There was a statistically significant difference in beam-on times between VMAT and all of the two arc RAD setups. With the two arc RAD setup with the balanced setting, the median decrease was between 7 and 17 s compared to the VMAT plan. On the static setting beam on time decreased with the three and four STAMP setups but slightly increased with the five STAMP setup. The static dominant setting caused the median beam on time to increase on the four and five static port setups by 4 and 15 s, respectively, compared to VMAT. However, even with the static dominant setting, delivery time decreased with the three STAMP setup by 15 s. All of the three arc plans with three STAMPs were faster than the associated VMAT plan with a median beam on time of 119 s and the median difference in beam on time was 11 s (*p* < 0.05). Even with an additional fourth static port, beam on time decreased in 7 of the 10 plans and the median beam on time was 122 s.

In breast N+ cases, heart *D*_*mean*_ decreased by 0.1–0.4 Gy in 53 of the 99 RAD plans compared to VMAT. Heart *V*_*16Gy*_ was very low in all plans but increased by 0.1–0.3 pp. in 28 RAD plans (Supplementary Material Table S6).

As in the breast N0 cases, ipsilateral lung mean dose and *V*_*16Gy*_ were significantly lower (*p* < 0.05) for breast N+ cases with VMAT compared to most RAD setups (Supplementary Material Table S7). The highest *D*_*mean*_ increases were seen with the static dominant setting. The highest median D_mean_ of 8.7 Gy and V_16Gy_ of 21.5–21.8% were in the three arc RAD plans. Small increases in ipsilateral lung *V*_*4Gy*_ were seen from VMAT to RAD. The ipsilateral lung *V*_*4Gy*_ was highest in the two three arc plans at 46.5% and 46.7% which were significantly higher (*p* < 0.05) compared to all two arc setups.

The RAD plans had slightly lower contralateral lung *D*_*mean*_ (Fig. 2C) compared to VMAT in breast N+ patients. The three arc three STAMP setup had the lowest median contralateral lung mean dose, also compared to all two-arc setups (*p* < 0.05). With two arcs, the lowest median contralateral lung *V*_*4Gy*_ (Fig. 3C) of 1.4% was achieved with the five STAMP plans using the static or static dominant setting. While, in the three arc plans *V*_*4Gy*_ was even lower at 1.1% and 0.8% with three and four STAMPs, respectively.

**Fig. 5:**
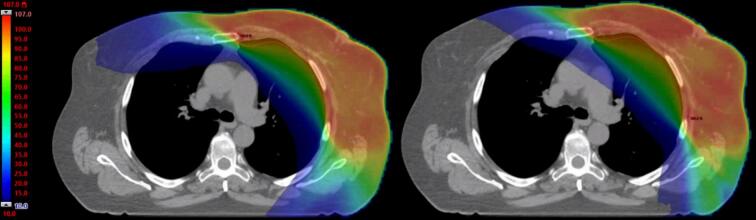
Images show the color wash of 10% to 107% of prescribed dose for a breast N+ case. The left image is from a VMAT plan, and the right image is from a two arc four STAMP RAD plan. The images show the difference in low dose spread between the two methods.

With RAD the median decrease in contralateral breast mean dose in breast N+ cases was between 1.1 and 2.2 Gy compared to VMAT (Fig. 2D & 5). The two arc four STAMP plans had a median *D*_*mean*_ of 4.3 Gy while for the six STAMP plans it was 3.8 Gy. The three arc plans and two arc static dominant plans had median contralateral breast mean doses of 2.7–3.0 Gy. With the different RAD setups median contralateral breast *V*_*4Gy*_ was between 18.5 and 27.5% which was significantly lower (*p* < 0.05) compared to VMAT at 47.9% (Fig. 3D). Increasing the STAMP weighting from balanced to static decreased the median *V*_*4Gy*_ spread from 26.7% to 19.0% and from 27.5% to 18.9% with four and five STAMPs, respectively. Changing the weighting from static to static dominant did not lower the volume notably. The three arc plans had a median *V*_*4Gy*_ of 19.5% and 18.8%.

Unlike in the N0 cases, in the N+ cases changes in body *V*_*20Gy*_ were smaller with the median change being −0.9% to 2.6% from VMAT to RAD (Supplementary Material Table S10). The median *V*_*20Gy*_ was lowest with the two arc five static port plans on the static dominant setting. Body *V*_*4Gy*_ decreased in 81 out of 99 RAD plans compared to VMAT. The median decrease in body *V*_*4Gy*_ was between 5.6% and 10.4% from VMAT to RAD.

The median beam on time of VMAT for the breast N+ plans was 139 s (Fig. 5B & Supplementary Material Table S5). All RAD plans with the balanced setting had lower beam on times than VMAT except for two plans with six static ports. The median difference between the balanced plans and VMAT was 25 s (*p* < 0.05), 18 s (*p* < 0.05) and 10 s (*p* < 0.05) with four, five and six STAMPs, respectively. With the static setting seven of the eleven plans had lower beam on times with four STAMPs but with five STAMPs only three RAD plans were faster. With four STAMPs when utilizing the static dominant setting, seven plans were between 1 to 16 s longer and the remaining four were 2 to 7 s faster than VMAT. In all plans five STAMPs with the static dominant setting caused RAD to be 8 to 44 s slower than the VMAT plan (*p* < 0.05). The median decrease between VMAT and the three arc RAD plans was 15 s (*p* < 0.05) and 11 s (*p* < 0.05).

## 4 Discussion

Comparable PTV_in_ coverage was achieved between RAD and VMAT in this study. RAD allowed more effective contralateral sparing, whereas ipsilateral lung doses were slightly lower with VMAT. The shortest beam on times were achieved with RAD, especially using the balanced setting in optimization. On the other hand, RAD plans required more MUs than VMAT plans.

Multiple different groups have compared different breast cancer radiotherapy techniques to HVMAT techniques [14], [17], [18], [20], [21], [22]. Lin et al. [22] compared the beam on times of forward planned IMRT, VMAT and HVMAT and found that VMAT had the shortest beam on time of 1.26 ± 0.01 min compared to 1.60 ± 0.18 min and 1.75 ± 0.07 min with IMRT and HVMAT, respectively. Aly et al. [18] used a single VMAT arc along with tangential IMRT fields in breast cancer cases with simultaneous integrated boost (SIB) and reported reductions in lung, heart and contralateral breast doses compared to VMAT. They also reported a slight reduction in treatment time with the hybrid method compared to traditional two arc VMAT (2.8 ± 0.5 min vs 2.9 ± 1.5 min). Jöst et al. [17] reported reductions in heart and ipsilateral lung doses between 3D-CRT and HVMAT while contralateral lung doses remained similar. They also reported an average treatment time of 92.0 ± 8.6 s with the hybrid technique compared to 84.7 ± 7.4 s with 3D-CRT. Comparing these results to the findings of this study, shows that Lin et al. and Jöst et al. had faster beam on times than the times measured in this study while Aly et al. reported longer beam on times. Clark et al. [24] reported delivery times that were faster than the times measured in this study. Their beam on times were 93 s for a two arc VMAT and 82 s for a one arc RAD plan with four STAMPs on the balanced setting for a SIB breast cancer case. Multiple groups [18], [21], [22] have reported slightly lower ipsilateral and contralateral lung mean doses, along with lower heart and contralateral breast doses, with HVMAT compared to VMAT. As with HVMAT, RAD was found to increase sparing of the contralateral lung and breast compared to VMAT. Unlike with HVMAT, the RAD setups utilized in this study increased ipsilateral lung dose in most of the treatment plans.

Individual clinics can have very different OAR priorities for VMAT planning which is acknowledged as a limitation of the study. The field setup solutions utilized in this study represent a variety of different number of STAMPs and the different STAMP weighting as well as number of arcs, which should serve as good starting points for users. For complex breast N+ cases, more complex setups than those used in this study could potentially achieve further OAR sparing with RAD. More field setup solutions should thus be studied in the future to find an optimal solution for breast N+ irradiation. Working on this article was started during the development of RapidArc Dynamic and the collimator rotation speed was set to 9 deg./s. This was changed to 15 deg./s in the released version which may cause slight differences in OAR parameters. Additionally, it is acknowledged that gamma analysis was not performed on all plans due to a technical issue that prevented us from taking the integrated dosimetry images with the EPID.

In conclusion, the findings show that RAD helps to conserve the contralateral side in breast radiotherapy. Based on the results, the two arc plans with three or four STAMPs would be recommended for N0 cases. The three STAMP variant showed no major penalty in beam on time when the weighting of the STAMPs was increased from balanced to static or static dominant. For simple N+ cases the two arc four STAMP plan with the balanced setting provides a good balance of increased contralateral breast sparing and beam on time. The three arc plans decreased the contralateral doses even further and thus the use of three arcs would be recommended for patients with poor DIBH or more complex anatomies. Overall, additional STAMPs had minor changes in plan quality.

## CRediT authorship contribution statement

**Topi Nykänen:** Writing – review & editing, Writing – original draft, Visualization, Methodology, Investigation, Funding acquisition, Formal analysis, Conceptualization. **Ville Raatikainen:** Writing – review & editing, Writing – original draft, Funding acquisition, Conceptualization. **Aarno Kärnä:** Writing – review & editing, Conceptualization. **Katariina A.H. Näkki:** Writing – review & editing. **Tuomas Koivumäki:** Writing – review & editing, Supervision, Project administration, Conceptualization.

## Declaration of competing interest

The authors declare the following financial interests/personal relationships which may be considered as potential competing interests: Wellbeing Services County of Central Finland and Hospital Nova of Central Finland has a research collaboration with Varian Medical Systems and Topi Nykänen, Ville Raatikainen, Aarno Kärnä, Katariina Näkki and Tuomas Koivumäki have received honoraria support from Varian Medical Systems.
