# Supplementary material for: Optimization of field setup for organs at risk sparing and beam on time in breast radiotherapy planning for a new volumetric modulated arc therapy based treatment technique
Source: Phys Imaging Radiat Oncol. 2026 Jun 7;39:101016. doi: 10.1016/j.phro.2026.101016 (PMC13284500; doi:10.1016/j.phro.2026.101016)
Supplement: Supplementary file 1 — Supplementary material [file mmc1.pdf]

Supplementary Material A

Planning methodology

Dose objectives – left-sided breast treatments

Table S1 Dose objectives used to optimize the breast cancer treatment plans for the study. Here, PTV = Planning target volume, LAD = Left anterior descending, Contralat. = Contralateral

		Whole breast		Breast with lymph nodes	
**Organ/structure**	**Dose parameter**	**Dose objective**	**Dose constraints**	**Dose objective**	**Dose constraints**
PTV_{skin} (3 mm)	$V_{95\%}$	95%	90%	95%	90%
	$V_{90\%}$	98%	95%	98%	95%
	D_{max}		110%		110%
	$V_{107\%}$	< 1 cm^3	< 3 cm^3	< 1 cm^3	< 3 cm^3
Heart	D_{mean}	2 Gy	4 Gy	2 Gy	4 Gy
	V_{16Gy}	2%	5%	5%	15%
	D_{max}	< 20 Gy	< 40 Gy	< 20 Gy	< 40 Gy
LAD	V_{30Gy}	0%	2 %	0%	2%
	V_{20Gy}	1%	2%	1%	
	D_{mean}	5 Gy	10 Gy	5 Gy	10 Gy
Ipsilateral lung	D_{mean}	8 Gy	13 Gy	9 Gy	13 Gy
	V_{16Gy}	15%	20%	20%	35%
	V_{4Gy}	35%	50%	50%	65%
Contralat. lung	D_{mean}	1 Gy	2.5 Gy	1.5 Gy	2.5 Gy
Contralat. breast	D_{mean}	1 Gy	3 Gy	3 Gy	7 Gy
Spinal cord	D_{max}			15 Gy	38 Gy

Plan preparation

All plans used two to four split arcs. Arc length was varied depending on the size of the breast and the quality of deep inspiration breath-hold (DIBH). Arc length was increased if the breast was large or when DIBH was poor. Breast N0 plans were optimized with shorter arcs than breast N+. In most of the RapidArc Dynamic (RAD) plans arcs were shorter compared to the volumetric modulated arc therapy (VMAT) plans as the static angle modulated ports (STAMP) allowed this.

In the RAD plans STAMPs were placed approximately on the tangential to the breast. In the two arc six STAMP plans, the anterior static angles were placed at approximately angles 300°, 308°

and 316° for the four and three STAMP plans the angle at 316° was removed. In the posterior arc the static angles were placed at approximately at 123°, 131° and 139°. For the four arc plans either the STAMP at 131° or at 139° was removed. Plans with three STAMPs had the posterior static angle at 131°. In the optimization window there are five options that set the weighting between the static angles and the arc by setting the number of control points of the static angles. These options are: arc dominant (-2), arc (-1), balanced (0), static (+1) and static dominant (+2) of which the three latter ones were utilized phijin this study. The number of control points at each STAMP for the different settings: arc dominant 2, arc 14, balanced 26, static 39 and static dominant 51.

Table S2 Arc spans for all whole breast (N0) plans. In the VMAT and RAD 3 Arc plans the third and fourth arcs had the same arc spans in the opposite direction as the first and second arcs.

	**VMAT**		**RAD 2 ARC**		**RAD 3 ARC**	
	Posterior start	Posterior end	Posterior start	Posterior end	Posterior start	Posterior end
**B1**	179	85	75	179	160	80
**B2**	179	75	80	160	170	100
**B3**	170	85	90	170	160	90
**B4**	179	80	90	179	160	90
**B5**	179	90	90	160	160	90
**B6**	179	90	90	170	170	90
**B7**	179	85	90	179	160	90
**B8**	179	75	75	179	179	85
**B9**	179	80	75	179	170	85
**B10**	179	80	90	170	160	100
**Median**	179	83	90	175	160	90
**Min**	170	75	75	160	160	80
**Max**	179	90	90	179	179	100
	Anterior start	Anterior end	Anterior start	Anterior end	Anterior start	Anterior end
**B1**	25	290	290	25	15	290
**B2**	35	295	295	25	0	290
**B3**	25	290	295	0	0	295
**B4**	20	290	290	0	0	295
**B5**	0	295	295	0	0	295
**B6**	0	290	295	0	0	290
**B7**	25	290	295	0	0	290
**B8**	35	290	290	20	20	290
**B9**	20	290	290	30	0	290
**B10**	20	290	290	0	0	290
**Median**	23	290	293	0	0	290
**Min**	0	290	290	0	0	290
**Max**	35	295	295	30	20	295

Table S3 Arc span for all breast with nodes (N+) plans. In the VMAT and RAD 3 Arc plans the third and fourth arcs had the same arc spans in the opposite direction as the first and second arcs.

	**VMAT**		**RAD 2 ARC**		**RAD 3 ARC**	
	Posterior start	Posterior end	Posterior start	Posterior end	Posterior start	Posterior end
**B11**	179	60	75	179	179	85
**B12**	179	85	90	179	179	90
**B13**	179	75	80	170	179	80
**B15**	60	179	60	179	179	75
**B16**	170	80	80	170	170	90
**B18**	179	60	60	179	160	90
**B19**	179	85	75	179	179	90
**B20**	179	80	85	179	170	90
**B26**	179	85	85	170	170	85
**B27**	170	85	85	179	170	90
**B28**	179	60	80	179	179	90
**Median**	179	80	80	179	179	90
**Min**	60	60	60	170	160	75
**Max**	179	179	90	179	179	90
	Anterior start	Anterior end	Anterior start	Anterior end	Anterior start	Anterior end
**B11**	60	290	290	45	25	290
**B12**	45	290	290	35	30	290
**B13**	45	290	290	45	25	290
**B15**	60	290	290	60	45	290
**B16**	35	290	290	25	15	290
**B18**	60	290	290	60	35	290
**B19**	45	290	290	75	30	290
**B20**	45	290	290	45	30	290
**B26**	45	290	290	30	30	290
**B27**	25	290	290	35	25	290
**B28**	60	290	290	35	20	290
**Median**	45	290	290	45	30	290
**Min**	25	290	290	25	15	290
**Max**	60	290	290	75	45	290

Optimization aperture was set to minimize the overlap between the lung and target volume to minimize ipsilateral lung dose (Fig. S1). This was done by limiting the collimator jaw x-axis to overlap 2-6 cm the ipsilateral lung. The optimization aperture opening was limited to fit the planning target volume (PTV) on the tangential angles. In the anterior direction the jaw was allowed to be open so that the x-axis jaw opening was 18 cm at maximum. Collimator angles were also set up to angles that minimize ipsilateral lung volume in the optimization aperture. In the RAD plans the STAMP angles were utilized to set the optimization aperture opening and collimator angles at the STAMPs. All RAD plans utilized the dynamic collimator setting ‘optimize

between static angles’. This setting sets the collimator angle to angles decided by the user at the STAMP, otherwise the collimator rotates to optimize the PTV into the aperture. The collimator angle was set at every static angle manually before optimization.

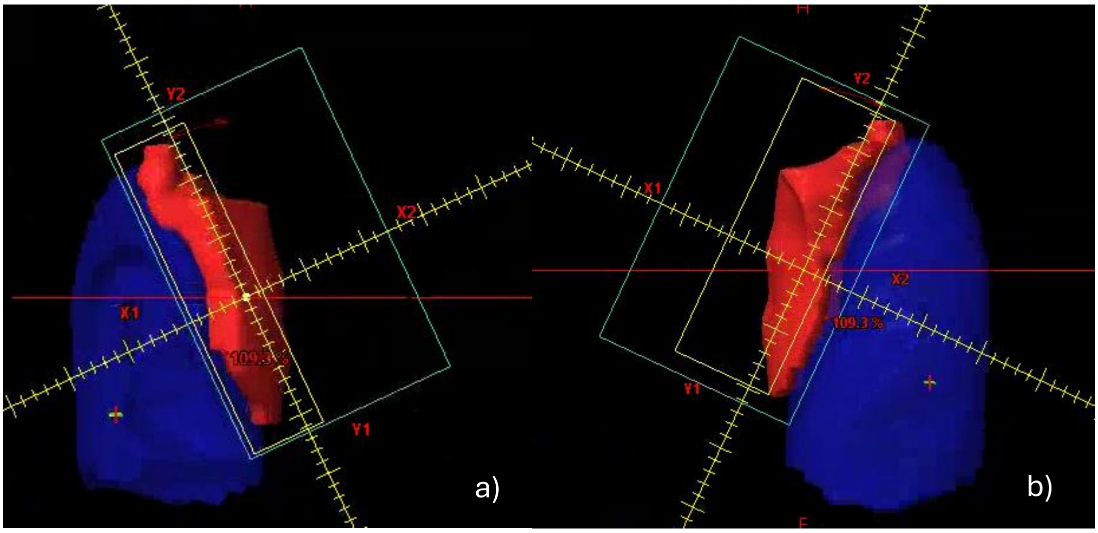

Fig. S1 Optimization aperture (light blue) was set up to minimize the ipsilateral lung volume in the aperture. Figure shows how the optimization aperture was set up on a two arc six STAMP plan of a breast with nodes case. a) Anterior arc b) Posterior arc

Adding the third arc

The third arc was added as a second anterior arc. One of the STAMPs was moved from the other anterior arc to the second arc. This enabled the dynamic collimator rotation to be controlled during gantry rotation by setting the collimator angle for the static angle while utilizing the ‘optimize between static angles’ -setting.

Optimization

Dose and normal tissue objectives are presented in figures S2-S3 and S5-S10. The plans were optimized with the highest priority being PTV coverage. The PTV objectives were set to 1.5 % over and under dosage of the prescribed dose. Priorities on the PTV were set to 170-210 on the upper and 180-210 on the lower objective. If needed, minima and maxima were removed from the initial plan with additional rounds of optimization.

#PTV vaRinta40Gy	1367.9						x
Upper	0.0	0.0	40.65	43.58	180		x
Lower	1367.9	100.0	39.45	28.51	190		x

Fig. S2 Optimization objectives for the PTV.

RapidArc dynamic plans utilized a separate structure for the automatic skin flash (Fig. S3).

PTV Flash RAD	509.2					x
Upper	0.0	0.0	40.65	43.43	160	x
Lower	509.2	100.0	39.45	29.18	180	x

Fig. S3 Optimization objectives for the separate skin flash structure.

A ring of 1.5 to 3.0 cm (Fig. S2) was utilized around the PTV to control the spreading of the high dose volume outside of the PTV.

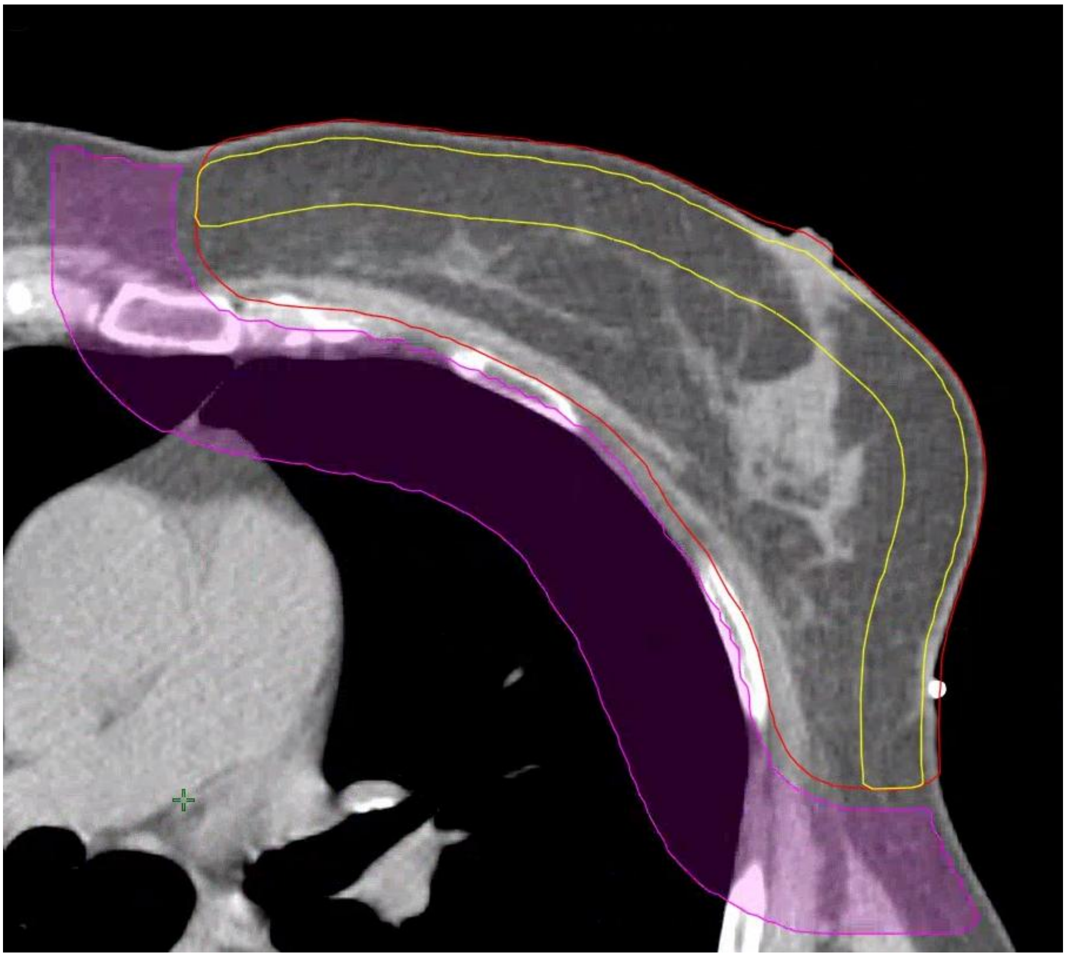

Fig. S4 Yellow structure was used as a separate structure for the skin flash optimization and the magenta ring structure around the PTV was used to control the spread of the high dose volume outside of the PTV.

PTV marg	1939.8					x
Upper	0.0	0.0	40.00	41.89	200	x
Upper	38.8	2.0	36.00	36.24	160	x

Fig. S4 Optimization objectives for the margin structure around the PTV.

During the optimization ipsilateral and contralateral lungs, heart and LAD were prioritized. The organs-at-risk (OAR) that overlapped with the PTV were cropped with a 3 mm margin to create optimization structures.

Right lung	3044.0						x
Mean			0.30	0.71	160		x
Left lung-PTV 3 mm marg	2648.5						x
Upper	906.0	34.2	2.23	7.19	120		x
Upper	633.5	23.9	5.54	12.35	120		x
Upper	291.1	11.0	15.02	23.81	120		x
Upper	67.0	2.5	25.06	32.87	120		x
Mean			6.50	7.90	140		x

Fig. S5 Optimization objectives for the contralateral (right) and ipsilateral (left) lungs. The ipsilateral lung was cropped by 3 mm for the optimization structure.

Heart	672.0					x
Upper	0.0	0.0	12.00	12.44	100	x
Upper	21.5	3.2	0.94	2.82	100	x
Upper	227.8	33.9	0.00	1.06	100	x
Mean			0.60	1.05	120	x

Fig. S6 Optimization objectives for the heart. The heart was cropped by 3 mm in cases where the PTV overlapped with the heart to create an optimization structure.

LAD	10.0					x
Upper	0.0	0.0	17.00	21.45	100	x
Upper	2.9	28.6	3.49	6.09	100	x
Upper	1.0	10.4	5.15	10.40	100	x
Upper	0.2	1.9	8.64	15.96	100	x
Mean			4.00	5.17	120	x

Fig. S7 Optimization objectives for the left anterior descending artery (LAD).

Other OARs were optimized with lower priorities to minimize the dose.

Medulla	55.1					x
Upper	0.0	0.0	15.00	7.36	80	x
Mean			0.80	1.24	80	x
Left humerus	39.4					x
Upper	0.0	0.0	30.00	21.36	80	x
Upper	18.9	47.9	1.94	4.18	80	x
Upper	3.9	9.9	5.00	10.78	80	x
Mean			4.50	5.44	80	x
Plexus	2.7					x
Upper	0.0	0.0	40.50	40.88	250	x

Fig. S8 Optimization objectives for the medulla, left (ipsilateral) humerus and brachial plexus. The left humerus was cropped by 3 mm in cases where the PTV overlapped with the heart to create an optimization structure.

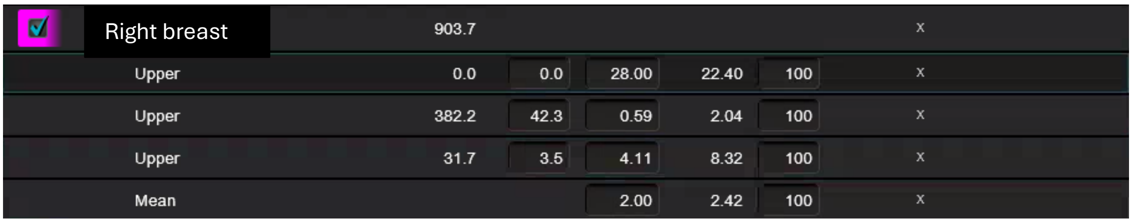

Fig. S8 Optimization objectives for the contralateral (right) breast.

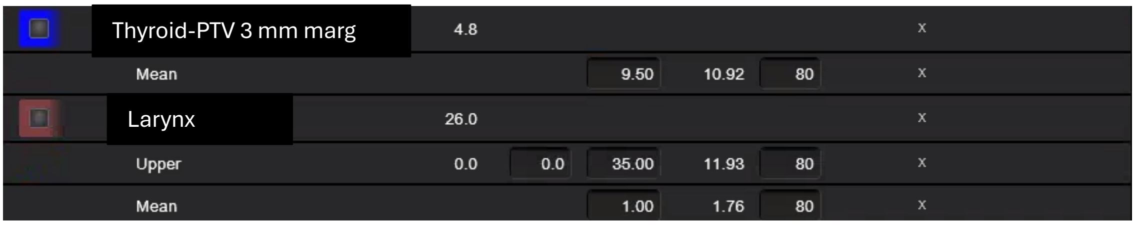

Fig. S9 Optimization objectives for the thyroid and larynx. In cases with lymph node irradiation the thyroid was cropped by 3 mm to create an optimization structure.

Plans utilized either automatic or manual NTO with a priority of 100-160. For manual NTO the settings below were found to be suitable for breast planning. The priority was set individually for each case.

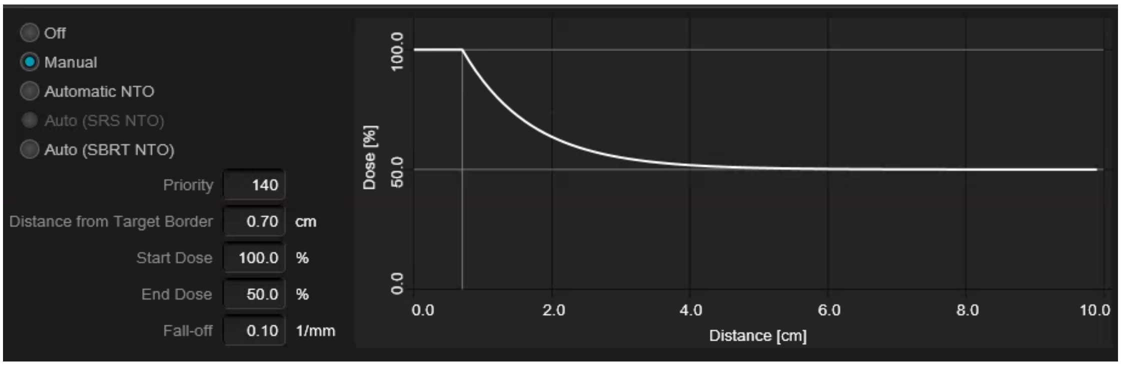

Fig. S10 Example normal tissue objective (NTO) parameters used in planning. The priority was varied approximately between 100-160 depending on the patient. Lower priorities were used for breast N0 cases.

Optimization times for six patients (3 N0 and 3 N+) were measured for VMAT and RAD. Timing was started the moment 'Start optimization' was pressed and stopped when Acuros was done with the final calculation. Convergence mode was set to 'On' on VMAT and no changes were made to the objectives during timing. All RAD plans were optimized with maximum iterations set to 1200. VMAT optimization took approximately 8.5 minutes whereas RAD optimization took approximately 1.3 minutes. These times depend on the hardware used as well as the optimization settings selected.

Dose volume histogram graphs

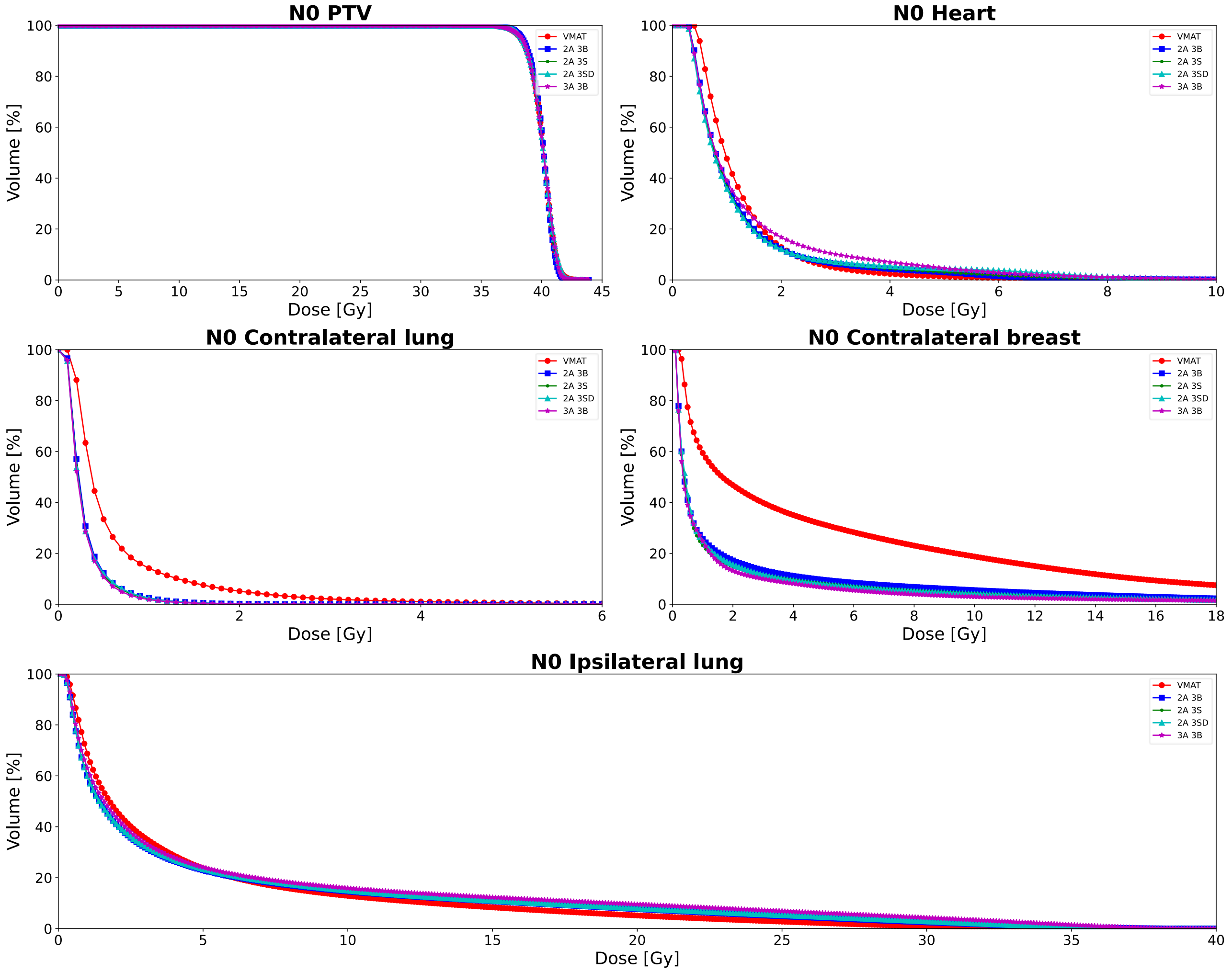

Fig. S11 Dose-volume histograms for OARs of a whole breast (N0) case for VMAT, all the two arc three STAMP (2A 3B, 2A 3S and 2A 3SD) plans and the three arc three STAMP plan (3A 3B). Planning target volume (PTV) Figure is from the evaluation PTV which has 3 mm of skin removed from the surface of the body. The figures for heart, contralateral lung and breast are cropped on x-axis for clarity.

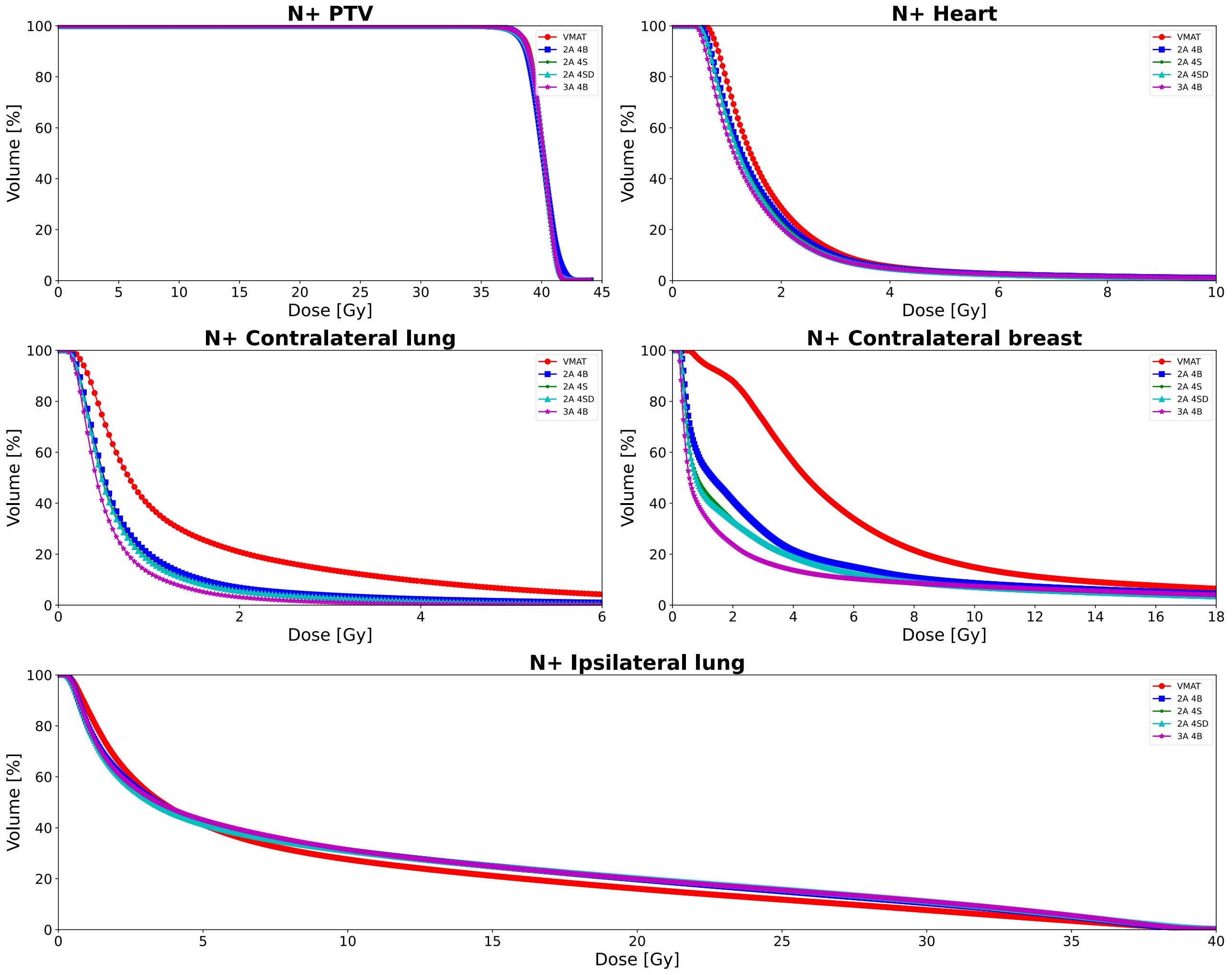

Fig. S12 Dose-volume histograms for OARs of a breast with nodes (N+) case for VMAT, all of the two arc four STAMP (2A 4B, 2A 4S and 2A 4SD) plans and the three arc four STAMP plan (3A 4B). Planning target volume (PTV) figure is from the evaluation PTV which has 3 mm of skin removed from the surface of the body. The figures for heart, contralateral lung and breast are cropped on x-axis for clarity.

Tables

Table S4 PTV_{in} $V_{95\%}$ and $V_{107\%}$ metrics median results for the Breast N0 and Breast N+ cases with range presented in square brackets. Here, xA=Arc with x being the number of arcs. B, S and SD refer to the static angle modulated port (STAMP) weighting B=Balanced, S=Static and SD=Static dominant with the number in front referring to the number of STAMPs.

	**PTV_{in}**	
	Breast N0	
Plan	$V_{95\%}$ (%)	$V_{107\%}$ (cc)
VMAT	96.3[95.5, 97.0]	0.0[0.0, 0.9]
2A 3B	96.5[95.5, 97.8]	0.0[0.0, 0.5]
2A 3S	96.5[95.5, 98.0]	0.0[0.0, 0.5]
2A 3SD	96.3[95.1, 97.5]	0.0[0.0, 0.2]
2A 4B	96.1[95.3, 97.8]	0.0[0.0, 0.2]
2A 4S	96.7[95.2, 97.8]	**0.0[0.0, 0.2]**
2A 4SD	95.9[95.2, 97.9]	0.0[0.0, 0.4]
2A 5B	95.9[95.2, 97.8]	0.0[0.0, 0.2]
2A 5S	96.2[95.3, 98.0]	0.0[0.0, 0.4]
2A 5SD	95.7[95.0, 97.8]	0.0[0.0, 0.5]
2A 6B	96.4[95.2, 97.8]	0.0[0.0, 0.1]
3A 3B	96.4[95.4, 97.2]	0.0[0.0, 0.3]
3A 4B	96.1[95.3, 97.9]	0.0[0.0, 0.8]
	Breast N+	
Plan	$V_{95\%}$ (%)	$V_{107\%}$ (cc)
VMAT	96.6[95.3, 98.2]	0.1[0.0, 0.4]
2A 4B	96.5[95.1, 97.8]	**0.0[0.0, 0.2]**
2A 4S	96.2[95.5, 97.7]	0.0[0.0, 0.3]
2A 4SD	96.6[95.6, 98.6]	0.0[0.0, 0.4]
2A 5B	96.9[95.0, 97.9]	0.0[0.0, 0.5]
2A 5S	97.0[95.7, 97.9]	0.0[0.0, 0.4]
2A 5SD	96.8[95.3, 98.1]	0.0[0.0, 0.4]
2A 6B	96.7[95.1, 97.5]	0.0[0.0, 0.8]
3A 3B	97.1[95.3, 97.9]	0.0[0.0, 0.5]
3A 4B	96.6[95.4, 98.2]	0.0[0.0, 0.6]

Bold font Denotes statistically significant difference between VMAT and RapidArc Dynamic ($p<0.05$)

Table S5 Median for the total Monitor units (MU) and beam on times for the VMAT and RAD treatment plans with the range presented in brackets. Here, xA=Arc with x being the number of arcs. B, S and SD refer to the static angle modulated port (STAMP) weighting B=Balanced, S=Static and SD=Static dominant with the number in front referring to the number of STAMPs.

Monitor units and Beam on time		
Breast N0		
Plan	MU	Beam on time (s)
VMAT	740[722, 882]	131[119, 137]
2A 3B	**989[912, 1046]**	***110[102, 119]***
2A 3S	**1014[927, 1098]**	***110[103, 126]***
2A 3SD	**994[918, 1103]**	**116[108, 132]**
2A 4B	**966[867, 1040]**	***110[101, 123]***
2A 4S	***1042[964, 1128]***	**125[111, 137]**
2A 4SD	***1072[956, 1149]***	***135[120, 148]***
2A 5B	**965[840, 1064]**	**120[101, 128]**
2A 5S	***1091[1009, 1208]***	***134[125, 153]***
2A 5SD	***1143[1062, 1217]***	***146[137, 171]***
2A 6B	**983[857, 1053]**	**122[113, 144]**
3A 3B	**987[885, 1120]**	**119[106, 134]**
3A 4B	**971[877, 1110]**	122[113, 144]
Breast N+		
Plan	MU	Beam on time (s)
VMAT	781[728, 878]	139[118, 150]
2A 4B	***939[857, 1095]***	***114[102, 129]***
2A 4S	***1043[967, 1230]***	*132[121, 146]*
2A 4SD	***1077[990, 1279]***	*142[129, 154]*
2A 5B	**953[820, 1077]**	**122[106, 136]**
2A 5S	***1116[974, 1259]***	*146[123, 157]*
2A 5SD	***1178[1049, 1329]***	***154[147, 182]***
2A 6B	**952[877, 1097]**	***126[118, 144]***
3A 3B	**972[914, 1070]**	***126[108, 142]***
3A 4B	**1005[939, 1089]**	**131[118, 146]**

Bold font Denotes statistically significant difference between VMAT and RapidArc Dynamic ($p<0.05$)
Italic font Denotes statistically significant difference between two arc and three arc 3 STAMP plans ($p<0.05$)
Underline Denotes statistically significant difference between two arc or three arc 3 STAMP and three arc 4 STAMP plans ($p<0.05$)

Table S6 Median D_{mean} and V_{16Gy} for heart with range presented in brackets. Here, xA=Arc with x being the number of arcs. B, S and SD refer to the static angle modulated port (STAMP) weighting B=Balanced, S=Static and SD=Static dominant with the number in front referring to the number of STAMPs.

Heart

Plan	D_{mean} (Gy)	V_{16Gy} (%)
Breast N0		
VMAT	1.3[0.8, 1.9]	0.0[0.0, 0.4]
2A 3B	1.2[0.7, 1.5]	0.0[0.0, 0.3]
2A 3S	**1.2[0.7, 1.6]**	0.0[0.0, 0.3]
2A 3SD	**1.1[0.7, 1.5]**	0.0[0.0, 0.3]
2A 4B	1.2[0.8, 1.5]	0.0[0.0, 0.2]
2A 4S	1.2[0.8, 1.6]	0.0[0.0, 0.3]
2A 4SD	1.2[0.7, 1.6]	0.0[0.0, 0.3]
2A 5B	1.2[0.8, 1.6]	0.0[0.0, 0.3]
2A 5S	**1.1[0.8, 1.5]**	0.0[0.0, 0.3]
2A 5SD	**1.1[0.7, 1.5]**	0.0[0.0, 0.3]
2A 6B	**1.1[0.8, 1.6]**	0.0[0.0, 0.1]
3A 3B	1.1[0.8, 1.7]	0.0[0.0, 0.2]
3A 4B	1.2[0.8, 1.9]	0.0[0.0, 0.2]
Breast N+		
Plan	D_{mean} (Gy)	V_{16Gy} (%)
VMAT	1.2[0.8, 2.5]	0.0[0.0, 1.3]
2A 4B	1.3[0.8, 2.4]	0.0[0.0, 1.5]
2A 4S	1.3[0.8, 2.4]	0.0[0.0, 1.6]
2A 4SD	1.3[0.8, 2.3]	0.0[0.0, 1.6]
2A 5B	1.3[0.7, 2.3]	0.0[0.0, 1.3]
2A 5S	1.2[0.8, 2.3]	0.0[0.0, 1.3]
2A 5SD	1.2[0.7, 2.3]	0.0[0.0, 1.4]
2A 6B	1.2[0.7, 2.3]	0.0[0.0, 1.6]
3A 3B	1.6[0.7, 2.3]	0.0[0.0, 1.4]
3A 4B	1.6[0.8, 2.3]	0.0[0.0, 1.6]

Bold font Denotes statistically significant difference between VMAT and RapidArc Dynamic (*$p<0.05$)*

Underline Denotes statistically significant difference between two arc or three arc 3 STAMP and three arc 4 STAMP plans (*$p<0.05$)*

Table S7 Median D_{mean} (Gy), V_{4Gy} (%) and V_{16Gy} (%) for Breast N0 and Breast N+ cases with range presented in brackets. Here, xA=Arc with x being the number of arcs. B, S and SD refer to the static angle modulated port (STAMP) weighting B=Balanced, S=Static and SD=Static dominant with the number in front referring to the number of STAMPs.

**Ipsilateral lung**			
Breast N0			
Plan	D_{mean} (Gy)	V_{4Gy} (%)	V_{16Gy} (%)
VMAT	4.3[3.5, 5.2]	27.2[19.1, 36.3]	7.8[5.2, 9.9]
2A 3B	<u>***4.7[3.8, 5.6]***</u>	26.2[21.2,34.1]	<u>***10.1[6.8, 13.1]***</u>
2A 3S	**4.7[3.7, 5.6]**	27.3[20.8, 34.1]	**10.2[7.2, 13.4]**
2A 3SD	<u>**4.7[3.8, 5.7]**</u>	<u>*26.4[21.1, 33.2]*</u>	**10.4[6.6, 13.5]**
2A 4B	**4.8[4.0, 5.9]**	27.0[21.8, 35.4]	**10.4[6.9, 13.4]**
2A 4S	**4.7[3.9, 5.7]**	26.4[21.2, 34.4]	**10.2[7.1, 13.5]**
2A 4SD	**4.8[3.9, 5.7]**	*26.4[21.1, 34.2]*	**10.5[6.7, 13.7]**
2A 5B	**4.9[3.7, 5.8]**	27.5[21.6, 32.9]	**10.6[6.2, 13.4]**
2A 5S	**4.9[3.8, 5.8]**	*27.3[20.3, 32.0]*	**10.6[6.7, 13.4]**
2A 5SD	**4.9[3.8, 5.8]**	*27.5[20.6, 33.3]*	**10.9[6.6, 14.1]**
2A 6B	**4.9[3.9, 5.9]**	27.7[20.6, 34.8]	**10.3[7.4, 13.6]**
3A 3B	**5.1[3.8, 5.9]**	28.9[20.3, 34.6]	**10.6[7.2, 13.5]**
3A 4B	**5.2[3.7, 5.8]**	28.7[20.3, 34.3]	**10.7[7.0, 13.4]**
Breast N+			
Plan	D_{mean} (Gy)	V_{4Gy} (%)	V_{16Gy} (%)
VMAT	8.4[6.6, 11.4]	44.9[38.7, 55.3]	20.0[13.8, 25.3]
2A 4B	<u>***8.5[7.1, 11.2]***</u>	<u>*45.7[40.7, 55.6]*</u>	***20.1[15.7, 28.2]***
2A 4S	<u>***8.4[7.1, 11.2]***</u>	<u>*45.7[40.7, 55.2]*</u>	<u>***20.2[15.9, 27.8]***</u>
2A 4SD	<u>**8.5[7.1, 11.1]**</u>	<u>*45.5[39.4, 53.8]*</u>	***20.5[15.7, 27.5]***
2A 5B	<u>***8.6[7.1, 11.5***</u>	<u>*45.5[40.0, 57.2]*</u>	**21.1[15.8, 29.5]**
2A 5S	<u>***8.5[7.1, 11.6]***</u>	<u>*45.3[39.5, 57.2]*</u>	**20.7[15.9, 29.0]**
2A 5SD	**8.5[7.2, 11.5]**	<u>*45.4[39.6, 55.8]*</u>	**20.6[16.3, 28.9]**
2A 6B	<u>***8.4[7.1, 11.4]***</u>	<u>*45.5[39.8, 55.7]*</u>	**20.4[15.5, 28.7]**
3A 3B	**8.7[7.2, 11.6]**	46.5[42.4, 56.7]	**21.5[15.6, 29.6]**
3A 4B	**8.7[7.2, 11.6]**	46.7[42.1, 56.4]	**21.8[15.8, 29.0]**

Bold font Denotes statistically significant difference between VMAT and RapidArc Dynamic (*$p<0.05$*)

Italic font Denotes statistically significant difference between two arc and three arc 3 STAMP plans (*$p<0.05$*)

<u>Underline</u> Denotes statistically significant difference between two arc or three arc 3 STAMP and three arc 4 STAMP plans (*$p<0.05$*)

Table S8 Median D_{mean} (Gy) and V_{4Gy} (%) for contralateral lung with range presented in brackets. Here, xA=Arc with x being the number of arcs. B, S and SD refer to the static angle modulated port (STAMP) weighting B=Balanced, S=Static and SD=Static dominant with the number in front referring to the number of STAMPs.

**Contralateral lung**		
Breast N0		
Plan	D_{mean} (Gy)	V_{4Gy} (%)
VMAT	0.5[0.3, 0.7]	0.0[0.0, 1.2]
2A 3B	***0.3[0.2, 0.5]***	0.0[0.0, 0.2]
2A 3S	***0.3[0.2, 0.5]***	**0.0[0.0, 0.5]**
2A 3SD	**0.3[0.2, 0.5]**	**0.0[0.0, 0.1]**
2A 4B	***0.3[0.2, 0.5]***	0.0[0.0, 0.3]
2A 4S	***0.3[0.2, 0.5]***	**0.0[0.0, 0.3]**
2A 4SD	***0.3[0.2, 0.5]***	0.0[0.0, 0.3]
2A 5B	***0.3[0.2, 0.5]***	**0.0[0.0, 0.4]**
2A 5S	***0.3[0.2, 0.5]***	**0.0[0.0, 0.3]**
2A 5SD	**0.3[0.2, 0.4]**	**0.0[0.0, 0.3]**
2A 6B	***0.3[0.2, 0.5]***	0.0[0.0, 0.6]
3A 3B	**0.3[0.2, 0.3]**	**0.0[0.0, 0.3]**
3A 4B	**0.3[0.2, 0.4]**	**0.0[0.0, 0.2]**
Breast N+		
Plan	D_{mean} (Gy)	V_{4Gy} (%)
VMAT	1.0[0.5, 2.5]	4.6[0.0, 23.4]
2A 4B	*0.8[0.5, 2.4]*	*2.9[0.2, 17.3]*
2A 4S	*0.7[0.5, 2.4]*	*2.0[0.1, 16.5]*
2A 4SD	***0.7[0.5, 2.2]***	*1.5[0.1, 15.5]*
2A 5B	***0.7[0.5, 2.4]***	*2.5[0.2, 17.9]*
2A 5S	***0.7[0.5, 2.2]***	1.4[0.1, 16.3]
2A 5SD	***0.7[0.5, 2.1]***	1.4[0.1, 14.9]
2A 6B	*0.8[0.5, 2.3]*	*2.9[0.1, 17.3]*
3A 3B	**0.6[0.4, 1.2]**	**1.1[0.0, 5.5]**
3A 4B	**0.7[0.4, 1.2]**	**0.8[0.0, 5.6]**

Bold font Denotes statistically significant difference between VMAT and RapidArc Dynamic ($p<0.05$)
Italic font Denotes statistically significant difference between two arc and three arc 3 STAMP plans ($p<0.05$)
Underline Denotes statistically significant difference between two arc or three arc 3 STAMP and three arc 4 STAMP plans ($p<0.05$)

Table S9 Median D_{mean} (Gy) and V_{4Gy} (%) for contralateral breast with range presented in brackets. From the Breast N+ cases two plans were removed from the data set due to bilateral mastectomy (*N*=9). Here, xA=Arc with x being the number of arcs. B, S and SD refer to the static angle modulated port (STAMP) weighting B=Balanced, S=Static and SD=Static dominant with the number in front referring to the number of STAMPs.

**Contralateral breast**		
Breast N0		
Plan	D_{mean} (Gy)	V_{4Gy} (%)
VMAT	2.6[1.1, 5.2]	20.8[4.6, 35.0]
2A 3B	<u>***1.7[0.6, 2.6]***</u>	<u>***10.2[2.1, 21.6]***</u>
2A 3S	***1.6[0.5, 2.8]***	**9.3[1.1, 24.3]**
2A 3SD	**1.6[0.4, 2.4]**	**8.8[0.2, 20.9]**
2A 4B	<u>***1.6[0.6, 2.7]***</u>	<u>**10.0[1.3, 23.9]**</u>
2A 4S	**1.5[0.5, 2.8]**	**8.5[0.8, 25.3]**
2A 4SD	**1.4[0.5, 3.1]**	**8.2[0.5, 30.0]**
2A 5B	<u>***1.8[0.7, 3.0]***</u>	<u>***10.0[2.3, 26.9]***</u>
2A 5S	**1.5[0.5, 2.7]**	**8.4[0.5, 24.2]**
2A 5SD	**1.5[0.4, 2.6]**	**8.5[0.4, 24.0]**
2A 6B	<u>***1.7[0.6, 3.3]***</u>	**9.7[1.1, 30.9]**
3A 3B	**0.9[0.4, 2.2]**	**4.4[0.7, 12.6]**
3A 4B	**0.9[0.5, 2.2]**	**3.9[0.4, 13.2]**
Breast N+		
Plan	D_{mean} (Gy)	V_{4Gy} (%)
VMAT	6.4[1.8, 8.1]	47.9[14.0, 66.4]
2A B SP4	**4.3[1.2, 7.0]**	**26.7[5.9, 50.4]**
2A S SP4	**3.3[1.2, 6.8]**	**19.0[6.1, 49.1]**
2A SD SP4	**3.0[1.2, 6.2]**	**19.0[5.8, 45.9]**
2A B SP5	**4.0[1.2, 7.1]**	**27.5[5.9, 56.3]**
2A S SP5	**3.1[1.1, 6.4]**	**18.9[5.8, 51.2]**
2A SD SP5	**2.8[1.0, 5.9]**	**18.5[5.4, 41.4]**
2A B SP6	**3.8[1.1, 6.8]**	**27.5[4.9, 49.6]**
3A B SP3	**2.7[1.1, 5.5]**	**19.5[4.9, 41.1]**
3A B SP4	**2.9[1.3, 5.1]**	**18.8[7.6, 35.2]**

Bold font Denotes statistically significant difference between VMAT and RapidArc Dynamic (*p<0.05)*
Italic font Denotes statistically significant difference between two arc and three arc 3 STAMP plans (*p<0.05)*
<u>Underline</u> Denotes statistically significant difference between two arc or three arc 3 STAMP and three arc 4 STAMP plans (*p<0.05)*

Table S10 Median V_{20Gy} (cm^3) and V_{4Gy} (cm^3) for the body structure with range presented in brackets. Here, xA=Arc with x being the number of arcs. B, S and SD refer to the static angle modulated port (STAMP) weighting B=Balanced, S=Static and SD=Static dominant with the number in front referring to the number of STAMPs.

Body

Breast N0		
Plan	V_{20Gy} (cm^3)	V_{4Gy} (cm^3)
VMAT	2581[1656, 3344]	4846[2833, 6205]
2A 3B	**2416[1643, 3181]**	**4165[2589, 6097]**
2A 3S	**2429[1635, 3157]**	**4094[2600, 5939]**
2A 3SD	**2433[1642, 3167]**	**4062[2563, 5844]**
2A 4B	**2432[1657, 3136]**	***4283[2621, 2832]***
2A 4S	**2410[1636, 3144]**	**4122[2597, 5818]**
2A 4SD	***2401[1617, 3131]***	**4106[2520, 5742]**
2A 5B	**2424[1651, 3163]**	***4241[2673, 6155]***
2A 5S	**2407[1640, 3152]**	**4125[2550, 5990]**
2A 5SD	**2389[1647, 3170]**	**4110[2502, 5916]**
2A 6B	**2400[1638, 3139]**	**4242[2591, 5963]**
3A 3B	**2410[1653, 3137]**	**4058[2647, 5204]**
3A 4B	**2399[1650, 3177]**	**4125[2555, 5321]**
Breast N+		
Plan	V_{20Gy} (cm^3)	V_{4Gy} (cm^3)
VMAT	2759[1773, 6044]	6099[4578, 12500]
2A B SP4	2635[1820, 5692]	**6068[4135, 12509]**
2A S SP4	2607[1779, 5636]	**6075[4172, 12457]**
2A SD SP4	2652[1785, 5575]	**5983[4134, 12106]**
2A B SP5	2696[1802, 5736]	**5949[4064, 12642]**
2A S SP5	2654[1776, 5623]	**5867[4116, 12298]**
2A SD SP5	*2602[1779, 5534]*	**5837[4136, 12014]**
2A B SP6	2646[1786, 5788]	**5884[4116, 12798]**
3A B SP3	2695[1717, 5820]	**6121[4352, 11288]**
3A B SP4	2629[1745, 5652]	**5983[4277, 11191]**

Bold font Denotes statistically significant difference between VMAT and RapidArc Dynamic ($p<0.05$)

Italic font Denotes statistically significant difference between two arc and three arc 3 STAMP plans ($p<0.05$)

Underline Denotes statistically significant difference between two arc or three arc 3 STAMP and three arc 4 STAMP plans ($p<0.05$)
